# Multidisciplinary approach to enhancing provider well-being in a metropolitan medical group in the United States

**DOI:** 10.1186/s12875-020-01323-6

**Published:** 2020-12-06

**Authors:** Lisa B. E. Shields, James T. Jennings, Joshua T. Honaker

**Affiliations:** 1 Norton Neuroscience Institute, Norton Healthcare, 210 East Gray Street, Suite 1102, Louisville, KY 40202 USA; 2 Norton Medical Group, Norton Healthcare, Louisville, KY USA

**Keywords:** Family practice, Primary care provider, Burnout, Well-being, Electronic health record

## Abstract

**Background:**

Physician burnout refers to depersonalization, emotional exhaustion, and a sense of lower personal accomplishment. Affecting approximately 50% of physicians in the United States, physician burnout negatively impacts both the physician and patient. Over a 3-year-period, this prospective study evaluated the multidisciplinary approach to decreasing provider burnout and improving provider well-being in our metropolitan community.

**Methods:**

A multidisciplinary Well-Being Task Force was established at our Institution in 2017 to assess the myriad factors that may play a role in provider burnout and offer solutions to mitigate the stressors that may lead to decreased provider well-being. Four multifaceted strategies were implemented: (1) provider engagement & growth; (2) workflow/office efficiencies; (3) relationship building; and (4) communication. Providers at our Institution took the Mayo Clinic’s well-being index survey on 3 occasions over 3 years. Their scores were compared to those of providers nationally at baseline and at 1 and 2 years after implementing organizational and individualized techniques to enhance provider well-being. Lower well-being index scores reflected better well-being.

**Results:**

The average overall well-being index scores of our Institution’s providers decreased from 1.76 at baseline to 1.32 2 years later compared to an increase in well-being index scores of physicians nationally (1.73 to 1.85). Both male and female providers’ average well-being index scores at our Institution decreased over the 3 years of this study, from 1.72 to 1.58 for males and 1.78 to 1.21 for females, while physicians’ scores nationally increased for both genders. The average well-being index scores were highest for providers at our Institution who graduated from medical school less than 5 years earlier (2.0) and who graduated 15–24 years earlier (2.3), whereas the average lowest scores were observed in providers who graduated ≥25 years earlier (1.37). Obstetricians/gynecologists and internal medicine physicians had the highest average well-being index scores (2.48 and 2.4, respectively) compared to other medical specialties. The turnover rate of our Institution’s providers was 5.6% in 2017 and 3.9% in 2019, reflecting a 30% decrease.

**Conclusion:**

This study serves as a model to reduce provider burnout and enhance well-being through both organizational and individual interventions.

## Background

Physician burnout is a work-related syndrome characterized by emotional exhaustion, depersonalization, and reduced sense of personal accomplishments that affects 50% of physicians in the United States [1–7]. Physician burnout is often associated with lower patient satisfaction and care quality, adverse patient health outcomes, higher medical error rates with an increased risk of malpractice, decreased work productivity, and increased provider turnover [4, 6–12]. Physicians who experience burnout have a lack of enthusiasm for work, high degrees of stress and fatigue, feelings of cynicism, and a greater likelihood of cardiovascular disease, insomnia, depression, strained relationships, alcohol and drug addiction, suicide, and shorter life expectancy [4, 8, 10, 13, 14]. Physicians have a 5-fold increased risk of suicide compared to non-physicians, accounting for 400 suicides annually in the United States [8]. Interestingly, it has been reported that physicians are significantly more likely than non-physicians to have tension with a co-worker, poor performance reviews, increased pressure, or fear of layoff that contributed to the suicide, reflecting an inability to cope with adversities related to their identity as a physician [15].

Approximately $4.6 billion in costs related to physician turnover and reduced clinical hours is attributable to burnout annually in the United States which represents approximately $7600 per employed physician each year [16]. In addition to the significant financial burden, the overwhelming administrative duties in part due to the electronic health record (EHR), stifling oversight by employers, loss of autonomy and control, and lack of colleague and organizational support contribute to provider burnout [7, 11, 14, 17–19]. It has also been reported that physicians are more likely to have symptoms of burnout and to be dissatisfied with work-life balance compared to working US adults [4].

The Stanford Medicine WellMD Center was created in 2015 “to advance the well-being of physicians and those they serve” [20]. Their leadership developed the WellMD Professional Fulfillment model that encompassed a (1) culture of wellness (shared values, behaviors, and leadership qualities that instill personal and professional growth, community, and compassion for self and others); (2) efficiency of practice (workplace systems, processes, and practices that promote safety, quality, effectiveness, positive patient and colleague interactions, and work-life balance); and (3) personal resilience (individual skills, behaviors, and attitude that contribute to physical, emotional, and professional well-being). Stanford established a validated survey that included perceived appreciation, personal/organization values alignment, peer supportiveness, perceived leadership support, control of schedule, EHR, experience, self-compassion, sleep-related impairment, and meaningfulness of clinical work [20]. The findings of the survey led Stanford to create an official peer support program, a separate house staff wellness survey, and personal resilience training courses.

A multidisciplinary Well-Being Task Force was established at our Institution in 2017 to investigate the factors that impacted provider burnout. We identified and implemented 4 multifaceted strategies derived from the Stanford Medicine WellMD Center and an internal survey at our Institution to reduce provider burnout and enhance well-being through both organizational and individual interventions: (1) provider engagement & growth; (2) workflow/office efficiencies; (3) relationship building; and (4) communication.

In the current study, we present our findings of average well-being index scores of our providers over a 3-year period compared to those of U.S. physicians nationally. Special attention focused on provider gender, years since medical school graduation, and medical specialty. We also describe how the numerous organizational and individual improvements implemented at our Institution positively impacted provider well-being.

## Methods

Under an Institutional Review Board-approved protocol, the current prospective study (January 1, 2017-December 31, 2019) was conducted in a metropolitan community consisting of 1476 providers (864 physicians, 563 advanced practice providers, and 49 other licensed professionals) as of March 1, 2020. Our Institution includes a Medical Group with more than 250 clinics, 4 adult hospitals, and a children’s hospital. A multidisciplinary Well-Being Task Force was established in July 2017 at our Institution to evaluate the various factors that may play a role in provider burnout and offer solutions to mitigate the stressors that may lead to decreased provider well-being (Table 1). The task force consisted of providers from different specialties (primary care and surgery), Advanced Practice Providers (APPs), leadership (Chief Medical Administrative Officer of our Medical Group), and leaders of our chaplains and outreach program. The electronic medical reporting system at our Institution is Epic.

**Table 1:** Goals of the Multidisciplinary Well-Being Task Force at our Institution

■ Obtain provider feedback to enhance the electronic health record and other operations	
■ Encourage provider engagement	
■ Improve provider well-being	
■ Ensure providers are supported	
■ Build constructive administration-provider relationships	
■ Develop self-care resources	
■ Obtain baseline provider well-being index scores	
■ Identify areas to implement targeted interventions	
■ Retest providers with the well-being index survey annually to determine yearly goals	

Prior to the development of this task force, an internal survey was conducted at our Institution in Spring 2017 that appraised providers’ sentiments pertaining to work-life balance and clinical/psychological/emotional burdens. The Engagement Survey, Epic After Hours Report, and Voice Gathering Sessions provided insight into work pressures that could be a source of burnout (Table 2). Providers who participated in the Engagement Survey were likely relatively-engaged and, therefore, provider burnout numbers may be higher when all providers were considered.

**Table 2:** Provider Internal Assessment Before Multidisciplinary Well-Being Task Force Implemented at our Institution

*Engagement Survey Data*	
○ 27% of provider respondents stated that they do lose sleep over work-related issues	
○ 37% of providers were not able to free their mind from work when they are away	
○ 42% reported being unable to disconnect from work during free time	
○ 45% not having the energy to pursue non-work activities after work day is over	
○ 38% overwhelmed by their work	
*Epic After Hours Report*	
○ On average a provider spends 43.65 min each work day in Epic outside of hours of 8:00 am-6:00 pm	
○ This finding means that a provider could be documenting in Epic for more than 5 h outside of work hours in a typical work week	
*Voice Gathering Sessions*	
○ “Conflicting with taking care of patients”	
○ “Dilution of care”	
○ “Work-life balance”	
○ “Psychological/emotional burdens”	
○ “Disconnection from administration”	

The Physician Well-Being Index Tool, a web-based tool developed by the Mayo Clinic for the purpose of evaluating overall well-being, was implemented at our Institution in July 2017. The Physician Well-Being Index accurately measures and tracks 6 dimensions of distress and well-being with a validated 9-question assessment [1, 21]. These dimensions included the following: likelihood of burnout, severe fatigue, suicidal ideation, quality of life, meaning in work, work-life integration, risk of medical error, dropout risk, and overall well-being [21]. Providers at our Institution were invited to participate in the anonymous provider well-being index survey through a link to access the survey sent to their Institutional e-mail addresses. Providers were able to download the well-being index mobile app to access the survey [22]. Our Institution obtained de-identified aggregate data. Since APPs at our Institution act as independent providers like physicians in most cases, the term “providers” in our study referred to physicians and APPs. The Well-Being Index Tool is a licensed survey instrument. Our Institution has a total of 4000 licenses to use, divided equally between physicians and APPs. The licenses are renewed annually.

Providers took the well-being index survey on 3 occasions over 3 years: (1) baseline – following establishment of multidisciplinary task force (7/1/2017–8/31/2017); (2) 1 year later (10/1/2018–12/15/2018); and 2 years later (10/15/19–12/31/19). Our providers’ average well-being index scores were compared to the national provider average for several metrics, including overall, by gender, by years since medical school graduation, and by medical specialty. Higher average well-being index scores indicate greater distress. The provider turnover rate was also presented.

The multidisciplinary Well-Being Task Force identified and implemented four multifaceted focus areas that play a significant role in decreasing provider burnout and enhancing well-being: (1) provider engagement & growth; (2) workflow/office efficiencies; (3) relationship building; and (4) communication (Table 3).

**Table 3:** Well-Being Strategic Plan Developed by Multidisciplinary Well-Being Task Force at our Institution

Focus Area	Activity	Detailed Description For Each Activity
*Provider Engagement & Growth*	> Leadership development and engagement	> Medical Director spends 25% of his time dedicated to provider development and well-being
	> Provider well-being champions	> 11 provider well-being champions
	> Employee Assistance Program (EAP) enhancement	> Contract with EAP provider
	> Well-being index survey	> Annually
	> Survey of burnout causes	> Annually
	> Connection interviews performed by medical directors and well-champions	> 20-30 minutes per interview, annually
*Workflow/Office Efficiencies*	> Epic optimization	> 1 hour per weekly meeting with 8-10 Medical Group leaders, 1 Epic builder, 4 trainers
	> Scribes	> Available to all providers at their own expense
	> Nursing staff assisting with inbasket	> 70-80 Licensed Practical Nurses
	> Automated prescription refill protocols	> Available to all providers
	> Advanced Practice Provider Onboarding	> On site of employment or change of department
	> Mentorship for MDs	> Ongoing for first 6-12 months of employment and as needed afterwards
*Relationship Building*	> NGaged program	> 4 times per year
	> Programs designed for specific provider groups	> 2-4 times per year depending on wants and needs of each group
	> Dinners for socializing with providers	> 2-3 times per year
*Communication*	> Well-being champions have in-person connections with all providers	> 20-30 minutes per interview, annually
	> Council meetings for all specialties	> Monthly
	> Share Well-Being Task Force initiatives and accomplishments with all providers	> Annually
	> Administrator/manager training includes importance of provider communication	> One occasion for a half-day training
	> Provider newsletter	> Minimal staff time required for coordination

### Provider Engagement & Growth

Several activities were implemented at our Institution to spur provider engagement and growth. A Provider Leadership Academy (PLA) was developed in 2012 to offer leadership training for providers. The Clinical Leadership Council (CLC) was established in 2013 with the aim of creating better provider leadership, engaging providers in well-being, and addressing patient concerns. Both the PLA and the CLC were created before the “intervention” period. However, the presence of provider leaders from the PLA and the voice gathering that the CLC provide allowed us to better craft a wellness program that met the needs of our providers. The Executive Medical Director Council was subsequently created in 2018. Well-being champions were selected among executive medical directors and general providers who serve as a voice and source of support. There are 6 service lines at our Institution, specifically primary care, cardiovascular, womens’ services, surgery, pediatrics, and behavioral health. Each service line had at least 1 well-being champion; larger service lines had up to 4 well-being champions. The different specialties at our Institution were all well-representated by well-being champions. Our Institution created a provider-only Employee Assistance Program (EAP) which offered confidential counseling and legal advice for providers with a separate location and phone number. There was a 400% increase in use by providers after we incorporated a separate facility and phone number.

### Workflow/office efficiencies

In response to providers’ frustration with the time-consuming Epic system, an Epic optimization team was developed to improve efficiencies in the EHR to decrease the time spent completing administrative obligations and increase clinical time. Practicing physicians were selected as Epic medical directors and trained to be Epic builders. An Epic (Verona) consultant was hired to evaluate for efficiencies and improve the Epic work flow, including documentation, prescription ordering, and reduction of unnecessary clicks. The Epic Signal Report presents the time that each individual provider spends performing in-basket functions, note writing, and chart review as well as documents whether this time is spent during or outside normal work hours. The Epic Signal Report serves as the standard to monitoring efficiency within the EHR system. To ensure that providers were most effectively using their clinical time and working at the top of their license, several aspects were incorporated such as scribes, nursing staff assisting with the inbasket, and automated prescription refill protocols that utilized a software platform to reduce the number of refills that the provider needed to approve. Advanced Practice Provider Onboarding was introduced in 2018 to provide educational resources, support, and delineation of roles and responsibilities for APPs at our Institution with the goal of enriching their quality of care and life. In 2020 we supplemented this process by adding a fellowship, mentoring program, and preceptorship for all new hires. This was an attempt to have better trained and more confident APPs when they enter the work force. All new hires went through the onboarding process in 2018–2019 and the enhanced program in 2020. Additionally, mentorship for new physicians at our Institution was available to respond to questions or concerns.

### Relationship building

The NGaged program was embraced as a means of encouraging work-life balance by offering opportunities for providers and their families to connect and attend events together in our community. Additionally, programs targeted to a particular group of providers were introduced such as formal lectures given by female leaders and social events designed for female providers. Dinners for socializing with providers were also encouraged. At any single event, approximately 30–40% of employed providers across the spectrum of medical specialties attended.

### Communication

The executive medical directors or other service line provider leaders conduct in-person connections with all providers at our Institution annually where the well-being champions bestow appreciative words to the providers. Council meetings were established for all medical specialties. Additionally, the initiatives and accomplishments of the Well-Being Task Force were shared with providers, and a provider newsletter was created. We have also reviewed administrative/manager training to impart the importance of provider communication.

## Results

The number of providers at our Institution who participated in the well-being index survey varied over the 3-year study period: 199 (22%) of the total 922 providers in 2017, 120 (12%) of the total 1010 providers in 2018, and 177 (15%) of the total 1158 providers in 2019.

### Percentage of providers at our Institution with high levels of distress compared to U.S. physicians nationally

A total of 41.29% of providers at our Institution had a well-being index score of ≥3 at baseline which is similar to that of U.S. physicians nationally (39.28%).

### Average provider overall well-being index scores at our Institution compared to U.S. physician average nationally

The average overall well-being index scores of our Institution’s providers decreased from 1.76 at baseline to 1.32 2 years later compared to an increase in well-being index scores of ≥14,900 U.S. physicians nationally (1.73 to 1.85) (Table 4).

**Table 4:** Comparison of Average Provider Well-Being Index Scores at our Institution to U.S. Providers Nationally

***A. Average overall well-being index scores of our Institution’s providers compared to U.S. physicians nationally***			
**Years**	**Our Institution’s providers**	**Sample Size**	**Physicians Nationally**
7/1/17–8/31/17 (Baseline)	1.76	199	1.73
10/1/18–12/15/18	1.402	120	1.73
10/15/19–12/31/19	1.32	177	1.85
***B. Average well-being index scores by gender of our Institution’s providers compared to U.S. physicians nationally***			
**Years**	**Our Institution’s providers**	**Sample Size**	**Physicians Nationally**
7/1/17–8/31/17 (Baseline)	Female: 1.78	121	2.19
	Male: 1.72	78	1.51
10/1/18–12/15/18	Female: 1.38	74	2.19
	Male: 1.68	43	1.51
10/15/19–12/31/19	Female: 1.21	123	2.24
	Male: 1.58	54	1.59
***C. Average well-being index scores by years since medical school graduation of our Institution's providers compared to U.S. physicians nationally***			
**Years**	**Our Institution’s providers**	**Sample Size (*n*** **= 199)**	**Physicians Nationally**
7/1/17–8/31/17 (Baseline)	< 5 years: 2.0	31	1.14
	5–14 years: 1.5	64	2.35
	15–24 years: 2.3	53	2.43
	≥ 25 years: 1.37	51	1.32
**D.** ***Average well-being index scores by medical specialty of our Institution’s providers***			
**Years**	**Our Institution’s providers**	**Sample Size (*n*** **= 199)**	
7/1/17–8/31/17 (Baseline)	Family medicine: 2.0	46	
	Internal medicine: 2.4	45	
	Obstetrics/Gynecology: 2.48	20	
	Pediatrics: 0.72	29	
	Surgical specialty: 1.83	29	

### Average provider well-being index scores by gender at our Institution compared to U.S. physician average nationally

Both male and female providers’ average well-being index scores at our Institution decreased over the 3 years of this study, from 1.72 to 1.58 for males and 1.78 to 1.21 for females, while providers’ scores nationally increased for both genders (Table 4). The male and female average well-being index scores were similar at baseline at our Institution (1.72 and 1.78, respectively), while the female average well-being index scores were lower than those for males at both 1 year later (1.38 versus 1.68) and 2 years later (1.21 versus 1.58).

### Average well-being index scores by years since provider medical school graduation at our Institution compared to U.S. physician average nationally

The average well-being index scores were highest for providers at our Institution who graduated from medical school < 5 years earlier (2.0) and who graduated 15–24 years earlier (2.3), whereas the average lowest well-being index scores were observed in providers who graduated ≥25 years earlier (1.37) (Table 4). The highest average well-being index score (2.43) was noted in U.S. physicians nationally who graduated from medical school 15–24 years earlier.

### Average well-being index scores by provider medical specialty at our Institution

Obstetricians/gynecologists and internal medicine physicians had the highest average well-being index scores (2.48 and 2.4, respectively) compared to other medical specialties. Pediatricians had the lowest average well-being index scores (0.72) (Table 4).

### Provider turnover rate at our Institution

The turnover rate was calculated as the number of providers who were no longer employeed by our Institution at the end of the year out of the total number of providers employeed by our Institution at the end of the year. The turnover rate of our Institution’s providers was 5.6% in 2017, 4.8% in 2018, and 3.9% in 2019, reflecting a 30% decrease from 2017 to 2019.

## Discussion

In a national sample of > 14,900 U.S. physicians, those with a physician well-being index score ≥ 3 were at greater risk for a number of adverse outcomes including a 2-fold higher risk of reporting a recent medical error, a 5-fold higher risk of burnout, 4-fold higher risk of severe fatigue, and 2-fold higher risk of suicidal ideation, and 3-fold higher risk of poor overall quality of life [22]. Our Institution had a representative population compared to the United States. Our Institution included 60% physicians and 40% APPs which was reflective of the whole nation. While we analyzed the data of the physicians across the nation in the study that included 14,900 physicians, our own data was also compared to the Well-Being Index scoring across the nation with both physicians and APPs, with a similar make-up to our own group. A total of 41% of providers at our Institution had a well-being index score of ≥3 at baseline which was similar to that of U.S. physicians nationally (39%). This striking number of physicians at our Institution and in the United States nationally who possess a high level of distress and numerous features of burnout corresponds closely to the approximately 50% of providers as reported in the literature [1, 4–6].

Following the implementation of the Well-Being Task Force at our Institution, the average overall well-being index scores of our Institution’s providers decreased from 1.76 at baseline to 1.32 2 years later compared to an increase in well-being index scores of U.S. physicians nationally. The myriad tactics ranging from Epic optimization, leadership engagement, onboarding, the NGaged program to heightened communications between providers and well-being champions most likely all contributed to this decline.

### Provider gender

Several studies have reported that female providers have a 20–60% increased odds of burnout [4, 5, 7, 23]. In Houkes and colleagues’ self-reported questionnaires of 340 general practitioners, burnout in men was primarily associated by depersonalization, while emotional exhaustion was most likely to cause burnout in women [23]. These authors speculate that men choose avoidance and withdrawal coping strategies whereas women become exhausted but do not depersonalize. In Shanafelt and colleagues’ survey of 7288 physicians, female physicians were more likely to be dissatisfied with work-life balance compared to their male colleagues (*p* = 0.002) [4]. Both male and female providers’ average well-being index scores at our Institution decreased over the 3 years of this study while providers’ scores nationally increased for both genders. The male and female average well-being index scores were similar at baseline at our Institution whereas the female average well-being index scores were lower than those for males at both 1 year later and 2 years later. These findings may particularly reflect the impact of focused efforts made at our Institution to target a specific provider group such as formal lectures given by female leaders and social events designed for female providers.

### Years since medical school graduation

It has been reported that younger physicians are at an increased risk of burnout symptoms [4], with those < 55 years old at more than double the risk of those > 55 years old [7]. In Del Carmen and colleagues’ survey study of 1774 physicians in 2014 and 1882 physicians in 2017, early career physicians (≤10 years since training) were more susceptible to burnout (odd ratio, 1.36), while physicians in their late career (> 30 years since training) were less vulnerable (odds ratio, 0.59) [10]. Our current study corroborates these findings as the highest average well-being index scores were highest for providers both at our Institution and nationally who graduated from medical school 15–24 years earlier. We also noted a peak in providers’ average scores who graduated from medical school < 5 years earlier and the lowest average scores in those who graduated ≥25 years earlier. The two provider groups that were at the highest risk for burnout warrant particular attention and intervention. Onboarding engagement may benefit providers who graduated from medical school < 5 years earlier, while Epic optimization, enhanced communication between administrators and providers, and well-being champions who serve as support personnel for providers in need may be valuable for providers who graduated from medical school < 5 years earlier and 15–24 years earlier.

### Provider medical specialty

In Shanafelt and colleagues’ survey of 7288 physicians, physicians practicing emergency medicine, general internal medicine, family medicine, neurology, or radiology had the highest risk of burnout, whereas dermatologists had a lower risk [4, 5]. Furthermore, physicians practicing dermatology, general pediatrics, and preventive medicine had the highest rated satisfaction with work-life balance, while physicians practicing general surgery, general surgery subspecialties, and obstetrics/gynecology had the lowest rates. These authors attribute the highest burnout rates to working primarily in the front line of access to care, except for pediatrics. Similar findings were encountered in the present study as obstetricians/gynecologists and internal medicine physicians had the highest average well-being index scores compared to other medical specialties, while pediatricians had the lowest average well-being index scores.

### Physician turnover rate

A high physician turnover rate not only may lead to diminished productivity, low morale, and diminished quality of patient care but also poses a financial burden. The costs of replacing a physician due to recruitment, onboarding, and lost patient care revenue equates to 2–3 times the physician’s annual salary [10, 12]. The turnover rate of our Institution’s providers was extremely low at 5.6% in 2017 and decreased by 30% to 3.9% in 2019.

### Causality between our implemented strategies and their perceived impact

Our internal evaluation prior to the initiation of the 4 strategies (Table 2) indicated that many of our providers were overwhelmed by their work, in particular the large of amount of time spent using Epic outside of work hours. Providers were frustrated by this extraordinary time commitment and lack of efficiency. The lack of work-life balance resulted in sleep loss, diminished energy to enjoy non-work activities, and psychological/emotional burdens. Additionally, there was deficient communication between providers and administration. Combining the feedback from our internal survey as well as Stanford’s and the Mayo Clinic’s established techniques of burnout reduction, we developed our 4 strategies. To address the Epic concern and to improve workflow/office efficiencies, Epic optimization was implemented which allowed providers to spend less time using the EMR during and after office hours and permitted more face-to-face contact with patients. Providers were less frustrated and more resilient and fulfilled, leading to enhanced provider-patient relationships, less administrative burden, and decreased burnout. The APP Onboarding led to happier providers, as reflected by the increased retention over the 3 years of our study. To promote a work-life balance, relationship building through socialization allowed providers and administration to get to know each other in a relaxed, non-work atmosphere. They were able to address any problems or concerns, which resulted in more engagement and less burnout. To enhance the communication between providers and administration, well-being champions forged valuable connections with all providers across different medical specialties which decreased the likelihood of burnout.

The 4 strategies implemented at our Institution were a staged-approach and did not need to be implemented as a package. Communication was the most important strategy as it bolstered trust among providers and administration/managers. The other three strategies built upon the foundation of communication. Workflow/office efficiencies were improved, and provider engagement/growth and relationship building were enhanced.

### Strengths and limitations

The 4 strategies in our study were fashioned from Stanford’s and the Mayo Clinic’s proven success that focused on professional fulfillment and avoidance of burnout. We applied their well-being techniques to the culture in our metropolitan community. Our 3-year study serves as a unique and effective model for incorporating numerous strategies aimed at decreasing provider burnout and boosting well-being in a metropolitan community. These approaches were directed at strengthening our Institution as a whole, replete with building a constructive administration-provider relationship, optimizing providers’ time by decreasing clerical burdens, and developing provider self-care resources to maintain a healthy work-life balance. Following the implementation of changes at our Institution, the well-being of providers at our Institution improved while that of the rest of the nation was either static or declined. The strong association between our implemented strategies and reduction in provider burnout suggests that our strategies substantially contributed to the decrease in provider burnout and improvement in provider well-being. As our approach to reduce provider burnout was based on Stanford’s well-recognized model, our beneficial tactics employed at our Institution in Kentucky may be generalized and applied to healthcare systems in other states.

Our study included providers in all fields of medicine with varied years since medical school graduation which permitted a comprehensive examination of factors that may lead to burnout. Providers were retested annually with the well-being index survey to determine yearly goals and areas for improvement based on provider feedback. The strategies implemented at our Institution led to a more efficient and standardized program that allowed providers to have more dedicated time to fulfilling their clinical and clerical obligations in a more relaxed atmosphere.

While the physician well-being index survey represents a valuable screening tool to improve physician self-awareness and identify physicians who may benefit from further evaluation or support [1, 24], our study only reported the average well-being index scores among providers at our Institution who completed the anonymous survey. In this respect, we were unable to specify the particular provider who may benefit from individualized attention. While we were incapable of identifying the specific providers who scored the highest on the well-being index survey, we hoped that the abundant modifications designed to bolster well-being at our Institution were advantageous for providers who were most at-risk for burnout. Another limiting factor in our work is the relatively low percentage of providers at our Institution who completed the 3 well-being index surveys. The response rates differed by specialties and by whether the respondents were physicians or APPs. There were different engagement levels depending the particular year. With continued dissemination of the strides made by the Well-Being Task Force to all providers at our Institution to combat provider burnout, we are encouraged that more providers will take the survey in the future. We also assume that there was self-selection bias of the providers who took the survey. An additional limitation was that the Engagement Survey was only performed before the initial well-being index survey. As there was an overlap in questions between these 2 surveys, the well-being index survey was the only one given in the follow-up period during our study. We subsequently re-implemented the Engagement Survey which is given concurrently with the well-being index survey.

Our current study is a timely contribution as the National Academy of Medicine proposed systemic changes in healthcare organizations, academic institutions, and all levels of government to create a positive work environment on October 23, 2019 [25]. The goal was to promote professional well-being, enhance patient care, reduce the risk of burnout, and balance job demands and resources [25]. Similar to our study, the proposal intends to regularly assess provider burnout.

## Conclusion

The epidemic of provider burnout has adverse repercussions not only on providers themselves but also on their patients, peers, and healthcare system. Individual providers and healthcare systems bear a mutual responsibility to address and eradicate the stressors that provoke burnout. The Hippocratic Oath, sworn by all new physicians, includes the phrase “may it be granted to me to partake of life fully and the practice of my art” [26]. Physician burnout, with its associated depersonalization, emotional exhaustion, and potential deleterious effects on one’s health, may impair or prevent a physician from adhering to this Oath. Rediscovering the joy and art of medicine while balancing work-life obligations is the ultimate goal. Further investigation is warranted into determining the organizational and individual provider interventions that may enhance provider well-being and mitigate the detrimental ramifications of provider burnout.

## Data Availability

The datasets used and analyzed during the current study are available from the corresponding author on reasonable request.

## Abbreviations

EHR: Electronic health record
APP: Advanced practice providers
CLC: Clinical Leadership Council
EAP: Employee Assistance Program
